# Plasma flow distal to tourniquet placement provides a physiological mechanism for tissue salvage

**DOI:** 10.1371/journal.pone.0244236

**Published:** 2020-12-21

**Authors:** Emily Busse, Cheryl Hickey, Nicole Vasilakos, Kennon Stewart, Fred O’Brien, Jessica Rivera, Luis Marrero, Michelle Lacey, Rebecca Schroll, Keith Van Meter, Mimi C. Sammarco

**Affiliations:** 1 Department of Surgery, Tulane School of Medicine, New Orleans, Louisiana, United States of America; 2 Department of Emergency Medicine, Louisiana State University Health Sciences Center, New Orleans, Louisiana, United States of America; 3 Department of Physiology, Tulane School of Medicine, New Orleans, Louisiana, United States of America; 4 Department of Mathematics, Tulane University, New Orleans, Louisiana, United States of America; 5 Orthopaedic Surgery Service, Dwight D. Eisenhower Army Medical Center, Fort Gordon, Georgia, United States of America; 6 Department of Orthopedics, Louisiana State University Health Sciences Center, New Orleans, Louisiana, United States of America; Medical University Innsbruck, AUSTRIA

## Abstract

Military literature has demonstrated the utility and safety of tourniquets in preventing mortality for some time, paving the way for increased use of tourniquets in civilian settings, including perioperatively to provide a bloodless surgical field. However, tourniquet use is not without risk and the subsequent effects of tissue ischemia can impede downstream rehabilitative efforts to regenerate and salvage nerve, muscle, tissue and bone in the limb. Limb ischemia studies in both the mouse and pig models have indicated not only that there is residual flow past the tourniquet by means of microcirculation, but also that recovery from tissue ischemia is dependent upon this microcirculation. Here we expand upon these previous studies using portable Near-Infrared Imaging to quantify residual plasma flow distal to the tourniquet in mice, pigs, and humans and leverage this flow to show that plasma can be supersaturated with oxygen to reduce intracellular hypoxia and promote tissue salvage following tourniquet placement. Our findings provide a mechanism of delivery for the application of oxygen, tissue preservation solutions, and anti-microbial agents prior to tourniquet release to improve postoperative recovery. In the current environment of increased tourniquet use, techniques which promote distal tissue preservation and limb salvage rates are crucial.

## Introduction

Civilian and military tourniquet use has been demonstrated to reduce the risk of death without increasing the risk of major limb complications [1, 2]. Such findings have encouraged increasingly widespread and liberal use of tourniquets in civilian patients including during limb surgery where tourniquets are routinely used to achieve a bloodless surgical field [3]. However, it is also widely known that prolonged tissue ischemia times and increased tourniquet pressure inversely correlates to tissue function and viability [4].

Recent studies in mice show a residual microcirculatory flow after tourniquet placement [5] and studies in pigs have shown that residual flow distal to the tourniquet results in improved locomotion in an acute limb ischemia model [6]. Both mice [5, 7] and pigs [6] are known to have extensive collateral vasculature that provide this residual flow while the tourniquet is in place. Theoretically, such residual blood flow continues to deliver oxygen to the distal tissue and could be exploited to deliver tissue sparing pharmacologics. However, there has not yet been a study that has identified and quantified this flow in humans, nor one that has demonstrated its utility *in vivo*.

The absence of a study on residual plasma flow dynamics in humans is due, at least in part, to the lack of readily available technology that can quantify residual blood flow distal to a tourniquet *in situ*. Plasma flow distal to the tourniquet in a mouse model is quantified using commercially available fluorescent compounds and near-infrared (NIR) small animal imaging, an approach limited, until recently, to small animals. Here we utilize the portable Fluobeam Imager designed specifically for clinic, in combination with the clinically available compound indocyanine green (ICG), to identify and quantify plasma flow distal to the tourniquet in pigs and humans. Further, we demonstrate the potential clinical utility of this residual flow in the mouse model by supersaturating the blood flow with oxygen in the ischemic limb, which significantly increased the tissue quality and decreased the intracellular hypoxia of the distal limb.

Together the confirmation of residual blood flow in humans using the clinical Fluobeam combined with a treatment-based mechanistic proof of concept in small animals has the potential to have a high impact on how residual blood flow may be used to decrease the risk of infection and transient or permanent injury to distal nerves, blood vessels, dermis, muscle, and bone in hospital settings.

## Materials and methods

### Ethics statement

All animal experiments were performed in accordance with the standard operating procedures approved by the Institutional Animal Care and Use Committee of Tulane University Health Sciences Center (Reference Number—336).

### In vivo mouse imaging studies

Eight-week old CD1 outbred mice (Charles River, Worcester, MA) were anesthetized with 1–5% isoflurane gas with continuous inhalation and the lower extremities were shaved and underwent a baseline scan using the IVIS XRMS imager (Perkin Elmer, Waltham, MA). We utilized a characterized mouse tourniquet model where an orthodontic rubber band is placed in the proximal thigh for four hours (240 min). Prior studies have utilized the rubber band tourniquet application with similar tourniquet times to assess locomotion and tissue quality [8–10]. At the end of the four-hour time period either 1 mg/kg ICG (Accutome, Malvern, PA) or Angiosense (Perkin Elmer, Waltham, MA), per manufacturer’s directions, was administered via tail vein injection with the tourniquet still in place. Mice were imaged immediately using the Perkin Elmer IVIS (ICG: 780 excitation/845 emission, Angiosense: 740 excitation/790 emission). The tourniquet was released, and mice were imaged again using the same parameters. Data was evaluated using Living Image 4.7.2 software (Perkin Elmer, Waltham, MA). For neutrophil activity Neutrophil Elastase 680 FAST (Perkin Elmer, Waltham, MA) was injected via tail vein injection, per manufacturer’s directions, 18 hours after tourniquet removal and imaged 24 hours after tourniquet removal to accommodate the optimal imaging window for reactivity and detection. Neutrophil Elastase 680 FAST is an activatable fluorescent compound that only identifies activated neutrophils capable of breaking down extracellular matrix. For Neutrophil Elastase FAST we utilized 640 excitation/710 emission. For quantification of ICG, Angiosense, and Neutrophil Elastase FAST raw radiant efficiency units were quantified using a grid overlay in conjunction with a constant circular region of interest (ROI) for each mouse. To standardize acquisition of average radiance circular ROIs were placed on the limb proximal and distal to the tourniquet in areas representative of only tissue in contact with the grid lines. N = 8 for plasma flow (N = 3 ICG and N = 5 Angiosense) and N = 8 for neutrophil elastase control and N = 10 for neutrophil elastase HBO.

### Hyperbaric oxygen treatment

HBO treatment of mice were as described previously [11]. The control group did not receive HBO treatment. Mice were placed in a hyperbaric chamber (Baromedical Research Institute–Van Meter and Assoc., Harvey, LA) and received 100% oxygen at 2.4 atmospheres absolute (ATA) during the last 90 minutes of tourniquet time (T = 150 min to 240 min). Compression and decompression of the chamber were executed at 2 psi/minute.

### Tissue collection and histology

Limb tissue samples for HBO-treated and control mice were harvested 48 hours after HBO treatment. Limbs were fixed in zinc-buffered formalin. Bone was decalcified and samples were processed for paraffin embedding and sectioned. Sections were stained with Hematoxylin and Eosin Y. Tissue was evaluated for quality using a Histologic Injury Severity Score (HISS) [12, 13] for the presence of myofiber damage, perivascular edema, erythrocyte extravasation, inflammatory infiltration, intraluminal thrombi, and loss of endothelium.

Intracellular oxygen changes were evaluated using Hypoxyprobe-1 Plus (Hypoxyprobe, Burlington, MA–see fluorescent immunohistochemistry for details) injected at 60 mg/kg prior to animal euthanasia and analyzed using histochemistry.

### Fluorescent immunohistochemistry

Immunofluorescent staining was performed on deparaffinized and rehydrated sections with Hypoxyprobe‐1 Plus (Hypoxyprobe, Burlington, MA, USA). Primary signal was amplified with either Alexa Fluor secondary antibodies (Thermo Fisher, Waltham, MA) or a tyramide signal amplification kit (Thermo Fisher, Waltham, MA) per manufacturer’s instructions and as previously described [11, 14].

### Statistical analysis

Statistically significant differences were determined using variations of one and two-way ANOVAs using SPSS Statistics (version 26) (IBM, Armonk, NY). A value of p<0.05 was deemed statistically significant. In all cases, data are represented as mean ± standard deviation (SD). One ICG mouse was identified as an outlier across the majority of ROI locations was therefore removed from analysis. Differences in total Histologic Injury Severity Scores for +T/+HBO, +T/-HBO, and -T/-HBO were analyzed with a Kruskal Wallis H-test. The Histologic Injury Severity Score for -T/-HBO was 0 for all samples and thus excluded from further distribution analysis. Distributions of +T/+HBO and +T/-HBO HISS were dissimilar, so a Mann-Whitney U test was used to compare mean ranks. Differences in neutrophil elastase radiant efficiency in the tourniquet and control leg either with or without hyperbaric oxygen (HBO) treatment were analyzed with a two-way ANOVA. No outliers were identified using studentized residuals greater than three standard deviations from the group mean. Neutrophil elastase raw pixel counts were transformed logarithmically to satisfy assumptions of the two-way ANOVA and all groups were determined to be approximately normally distributed via Shapiro Wilk (p>0.05). Main effect analysis included Bonferroni correction for multiple comparisons. Tourniquet treatment had no effect on neutrophil elastase activity so a one-way ANOVA was used to explore differences in HBO treatment.

Graphs were compiled using GraphPad Prism (version 7) (GraphPad, San Diego, CA).

### In vivo swine imaging studies

Four 70 kg Yorkshire swine were anesthetized using 2–8 mg/kg telazol and 1–3 mg/kg xylazine. A Combat Application Tourniquet (CAT) (CAT Resources, Rock Hill, SC) was placed on the left forelimb of the swine. Attainment of limb occlusion pressure (LOP) was confirmed by verifying the absence of arterial signal using Doppler ultrasound. Both the tourniqueted and control forelimbs were mounted to a stabilized board below a portable Fluobeam near infrared imager (Fluoptics, Cambridge, MA) positioned 21 inches from the target region of interest to include both limbs. 120 seconds of imaging was initiated and 5 mg ICG was injected at T = 20 sec to provide a baseline reading without ICG. A second 120 seconds of imaging immediately following the first session and the tourniquet was removed at T = 20 seconds. Data was analyzed using the Fluoptics software (Fluoptics, Cambridge, MA).

## Human imaging studies

Written ethical approval for this study was received from the Tulane School of Medicine Institutional Review Board (Reference Number—1119164). 24 volunteers were recruited after obtaining written informed consent. Data was stripped of personal identifiers for analysis purposes and the database did not include any information that could link back to individual subjects.

24 volunteers between the ages of 18 and 60 participated in the study. Vital signs were recorded prior to tourniquet placement: gender, age, weight, height, body mass index (BMI), systolic and diastolic blood pressure (SBP and DBP, respectively), pulse, respiratory rate, temperature, and oxygen saturation. A Combat Application Tourniquet (CAT) was placed on the non-dominant arm in the proximal humerus by a trained physician and tightened until there was no detection of arterial pulse by Doppler ultrasound. Volunteers were imaged palm down in the prone position with the Fluobeam imager positioned 21 inches from the target region of interest to include both limbs. Additional imaging was performed on one subject at 7 inches. 120 seconds of imaging was initiated and 5 mg ICG was injected at T = 20 sec to provide a baseline reading without ICG. Another 120 second session of imaging followed during which the tourniquet was removed at T = 20 seconds from initiation. Videos were recorded at 25 frames per second. For data analysis we reduced all videos to 1 frame per second for a total of 109 frames. Brightfield snapshots that are automatically initiated every 10 seconds by the Fluoptics software during recording were removed to eliminate artifactual readings and restrict analysis to the fluorescent data. The range of ICG intensities were thresholded in each frame and an average pixel intensity was generated for each frame over time. The initial 10 seconds of the first imaging session prior to ICG injection were averaged to adjust for background interference. The 10 seconds prior to tourniquet release was averaged and the 10 seconds after tourniquet release was averaged as a measure of plasma flow prior to and after tourniquet release, denoted by Preflow and Postflow, respectively. Flow values were analyzed on the logarithmic scale, with the difference in average flow defined as Difference in Log Flow (DLF) = log(Postflow/Preflow).

## Results

### Tourniquet placement does not completely occlude plasma flow in mice

**Fig 1:**
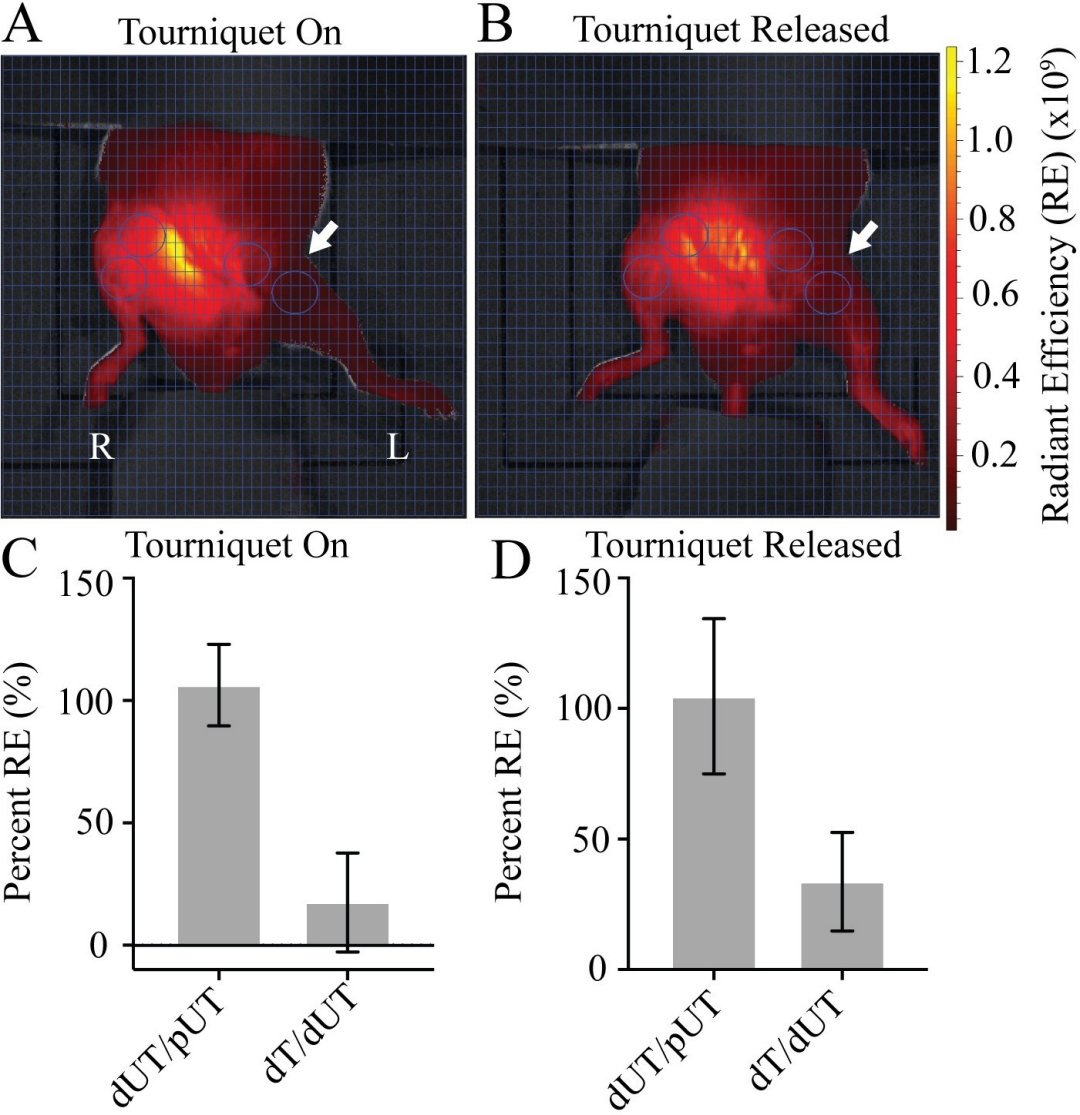
Mice demonstrate plasma flow distal to the tourniquet. **A)** Tourniquet placement (arrow) shows Angiosense (red) distal to the tourniquet in the left leg while the tourniquet is in place. **B)** Release of tourniquet restores flow to the distal limb. Region of interest (ROI—circles) target areas proximal and distal to the tourniquet in both tourniqueted left leg (L), and untourniqueted (R) right leg limbs. Ventral view. Fluorescent signal indicates Angiosense detection (red) in units of radiant efficiency (RE) (see scale bar). Representative images shown, N = 8. **C)** Plasma flow in the dUT/pUT and dT/dUT limbs with the tourniquet on. **D)** Plasma flow in the dUT/pUT and dT/dUT limbs after tourniquet released. N = 8. Error bars represent SD.

To evaluate plasma flow post-tourniquet in a mouse model we utilized the well-characterized orthodontic rubber band model [8–10] where an orthodontic rubber band is placed on the proximal thigh four hours. Plasma flow in the distal untourniqueted (dUT) limb was quantified as a percent of the proximal untourniqueted (pUT) limb (dUT/pUT) to account for differences in local distal and proximal vascularity (Fig 1A and 1B). As expected, plasma flow in distal UT limb was comparable to flow in the proximal UT limb (dUT/pUT). Distal flow in the tourniqueted limb (dT) after four hours of tourniquet was 17.59% as a function of the distal UT limb (dT/dUT) (Fig 1C). Predictably, removal of the tourniquet from the left leg showed no detectable impact on either the distal or proximal limb of the UT right leg (dUT/pUT). The distal tourniqueted limb after tourniquet release showed 33.69% flow when normalized to the dUT limb (dT/dUT) (Fig 1D).

### Tourniquet placement does not completely occlude plasma flow in swine

**Fig 2:**
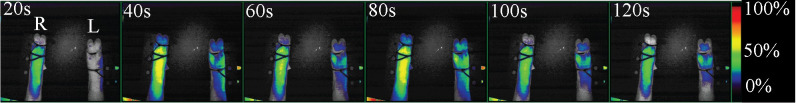
Pigs demonstrate plasma flow distal to the tourniquet. ICG signal is seen in both tourniqueted (left leg—L) and untourniqueted (right leg—R) distal limbs within 20 seconds of injection and increases over time. Ventral view. Fluorescent signal indicates ICG detection. Representative image shown, N = 4. Relative fluorescence by percent shown.

We then scaled this NIR technique for a large animal model by measuring injected ICG using the portable Fluoptics Fluobeam NIR clinical imager to identify plasma flow distal to the tourniquet in swine. A CAT was placed on the left forelimb of the swine and tightened until there was absence of arterial signal by Doppler ultrasound. Tourniquet placement reduced plasma flow to approximately 50% when normalized to the untourniqueted (UT) limb (Fig 2). ICG was detectable in both limbs after 20 seconds and the signal continued to increase over 120 seconds indicating that residual plasma flow distal to the tourniquet is evident in this large animal model.

### Tourniquet placement does not completely occlude plasma flow in humans

To determine whether plasma flow distal to the tourniquet extends beyond animal models and occurs in humans we enlisted 24 healthy subjects to evaluate plasma flow with tourniquet use using ICG and the Fluobeam imager. A CAT was placed on the non-dominant proximal arm of the volunteer and tightened until there was an absence of arterial signal using Doppler ultrasound in order to provide a metric ensuring that tourniquet application pressures were reproducible and consistent. Tourniquet remained in place for approximately 5 minutes, providing a length of time that was uniform and reasonably tolerated in humans [15]. Plasma flow was quantified in the distal tourniqueted limb as a function of the proximal tourniqueted limb (dT/pT). Overall, 70% of the subjects had measurable flow in the distal limb, with flow completely occluded in only 30%. 50% of volunteers had between 0.5% and 50% plasma flow, 12.5% of volunteers had 50% to 99% plasma flow, and 8.5% of the volunteers had 100% plasma flow distal to the tourniquet.

**Fig 3:**
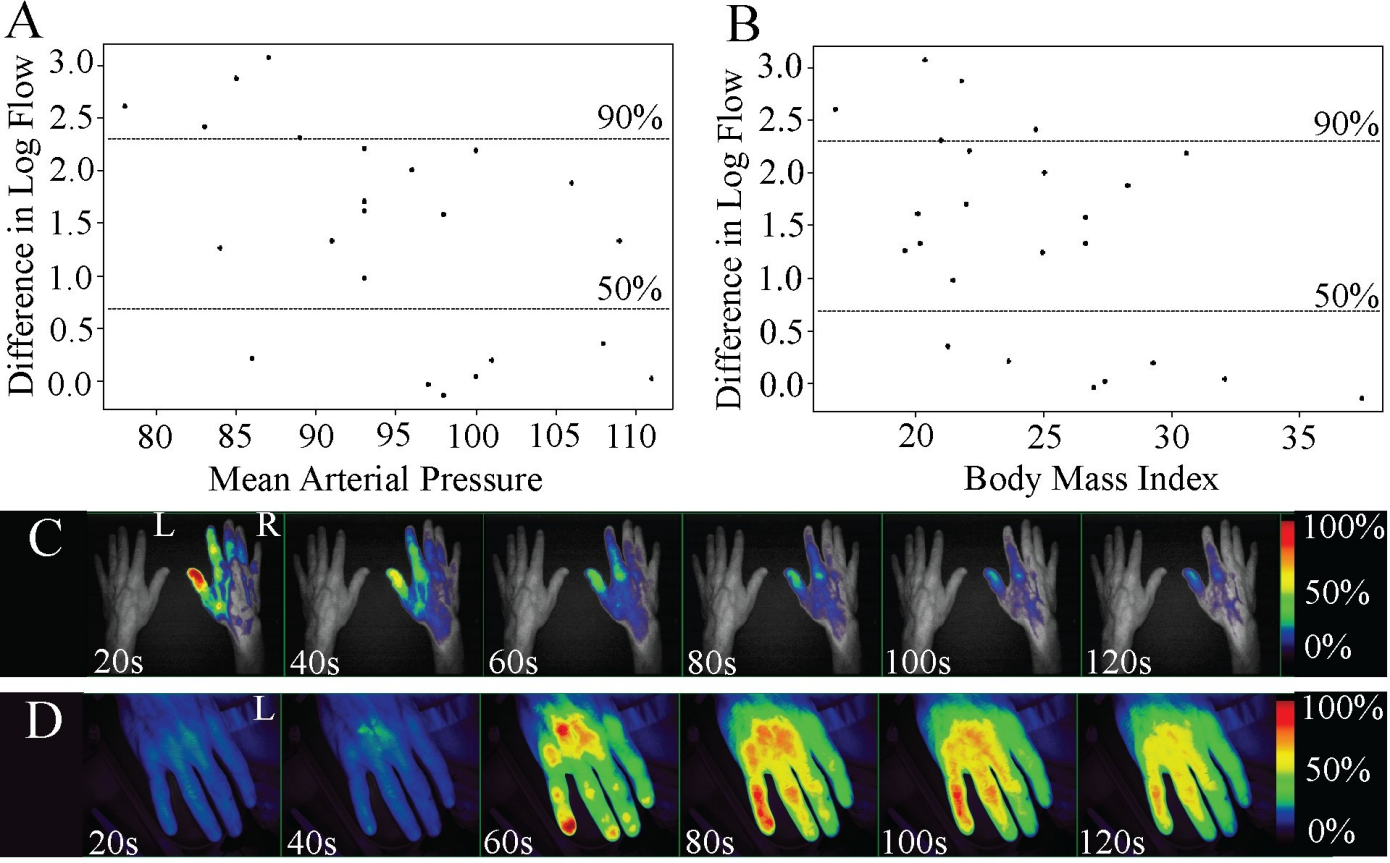
Plasma flow distal to the tourniquet in human subjects. Greater restrictions in flow during tourniquet were associated with **A)** lower MAP in the tourniqueted arm (r = -0.525) and **B)** lower BMI (r = -0.524). DLF of 90% or greater was significantly associated with lower MAP (*p* = 0.018) and lower BMI (*p* = 0.003). A subject who showed **C)** no detectable flow in the tourniqueted arm (L) with a 21-inch imaging distance demonstrated **D)** detectable ICG in the tourniqueted arm when the imaging distance was decreased to 7 inches. Dorsal view. Fluorescent signal indicates ICG detection. Relative fluorescence by percent shown.

For correlation with vitals we averaged the flow ten seconds after tourniquet release (Postflow) and ten seconds prior to tourniquet release (Preflow) to determine the difference in flow within the tourniqueted arm using the DLF log scale. Flow was detected in the fingertips initially, followed by proximal infiltration through the hand (S1 Video). Flow values were analyzed on the logarithmic scale, calculated as Difference in Log Flow (DLF) = log(Postflow/Preflow). Within the tourniqueted arm, DLF was negatively correlated with both increased mean arterial pressure (MAP), calculated as MAP = 1/3(SBP-DBP) + DPB and BMI (*r* = -0.525 and *r* = -0.524, respectively), supporting that those volunteers with increased MAP and BMI had increased flow prior to tourniquet removal. Furthermore, both the average MAP and BMI measurements for individuals with flow restricted by 90% or more were significantly lower compared to those with lesser flow restrictions (*p* = 0.0003 and *p* = -0.018, Student's t-test) (Fig 3A and 3B), also supporting the converse, that those with greater flow demonstrated a higher BMI and MAP. In addition, overall flow in the untourniqueted arm was also negatively associated with both MAP (r = -0.44) and BMI (r = -0.38). All groups were determined to be approximately normally distributed via Shapiro-Wilk test (p>0.05).

The design of the study was to incorporate both a test (tourniqueted) and control (untourniqueted) arm within the field of view for simultaneous, bilateral comparison. However, since increased imaging distance was required to accommodate both the control and test arm we expected a decrease in efficiency of excitation and consequentially emission and capture of ICG fluorescence. To test whether fluorescence intensity would increase in human subjects relative to lower imaging distance, we decreased the imaging distance by 2/3 on a single subject who showed little or no flow initially. A comparison of relative fluorescence from the dT limb confirms that decreasing the imaging distance improves detection of ICG (Fig 3C and 3D).

### Hyperbaric oxygen improves tissue quality and reduces hypoxia in tourniqueted mouse limbs

**Fig 4:**
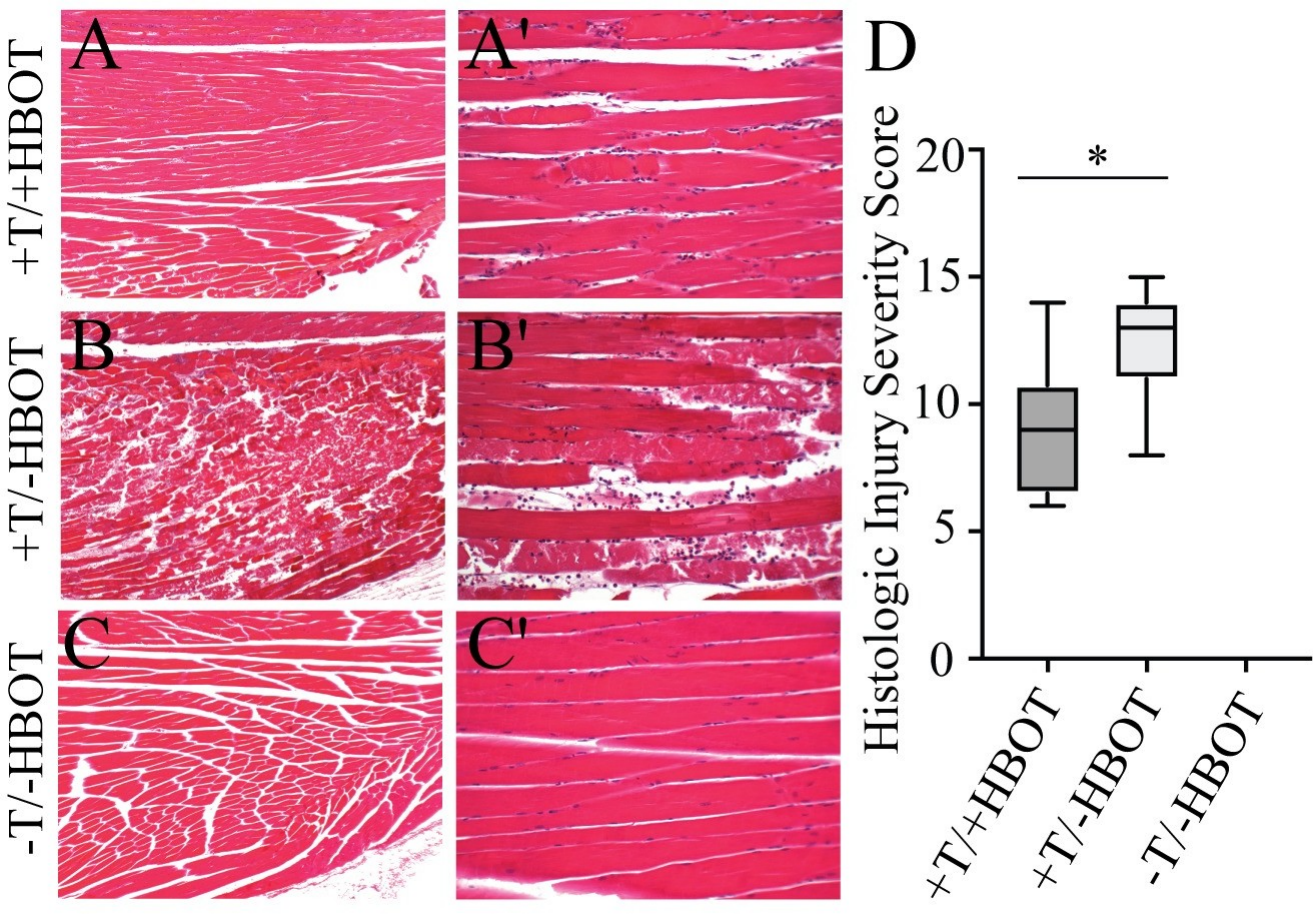
Neutrophil elastase activity and intracellular hypoxia in injured tissue treated with hyperbaric oxygen (HBO). H&E staining (40X) of tissue samples from +T/+HBO, +T/-HBO, and -T/-HBO (control tissue) muscle samples, taken 48 hours after treatment shows that tissue quality is markedly increased in **A)** tissue from mice that received HBO treatment, as compared to **B)** tissue from mice that received no treatment. **C)** Tissue from control mice that did not receive either a tourniquet or HBO treatment are shown for comparison. **A'-C')** H&E staining (100X) of tissue samples. **D)** Histologic Injury Severity Scores. Box and whisker plot shows highest and lowest HISS score, as well as quartile values.

We then wanted to test whether residual plasma flow distal to the tourniquet could be leveraged to improve tissue salvage techniques in the ischemic limb. To do this we utilized hyperbaric oxygen (HBO), which works through inhalation of high concentrations of oxygen in a pressurized chamber. Oxygen delivered via hemoglobin is limited by the carrying capacity of hemoglobin, which is optimally 20 mL O_2_/dL of blood. However the application of oxygen under pressure allows oxygen dissolved in the plasma to increase carrying capacity beyond hemoglobin limitations by approximately 22.5% [16]. To treat with HBO we applied 100% oxygen at 2.4 ATA for the last 90 minutes of the four-hour tourniquet placement in the mouse model. Mice received one treatment of hyperbaric oxygen while the tourniquet was in place and whole limbs were harvested 48 hours after tourniquet release. Histological analysis of the skeletal muscle tissue was performed using the HISS system, which showed that the application of HBO with a tourniquet in place resulted in a significant reduction in tissue damage (Fig 4A) when compared to the tourniqueted group without HBO (Fig 4B). Mean ranks were 5.81 and 11.19 for +T/+HBO and +T/-HBO, respectively, and this difference was statistically significant (p = 0.021). An untourniqueted control group that did not receive HBO is shown for comparison (Fig 4C). Comparison of total HISS between tourniquet groups support that treatment with HBO decreased or protected the tissue from injury. Data is shown as quartile ranges with minimum and maximum HISS values.

**Fig 5:**
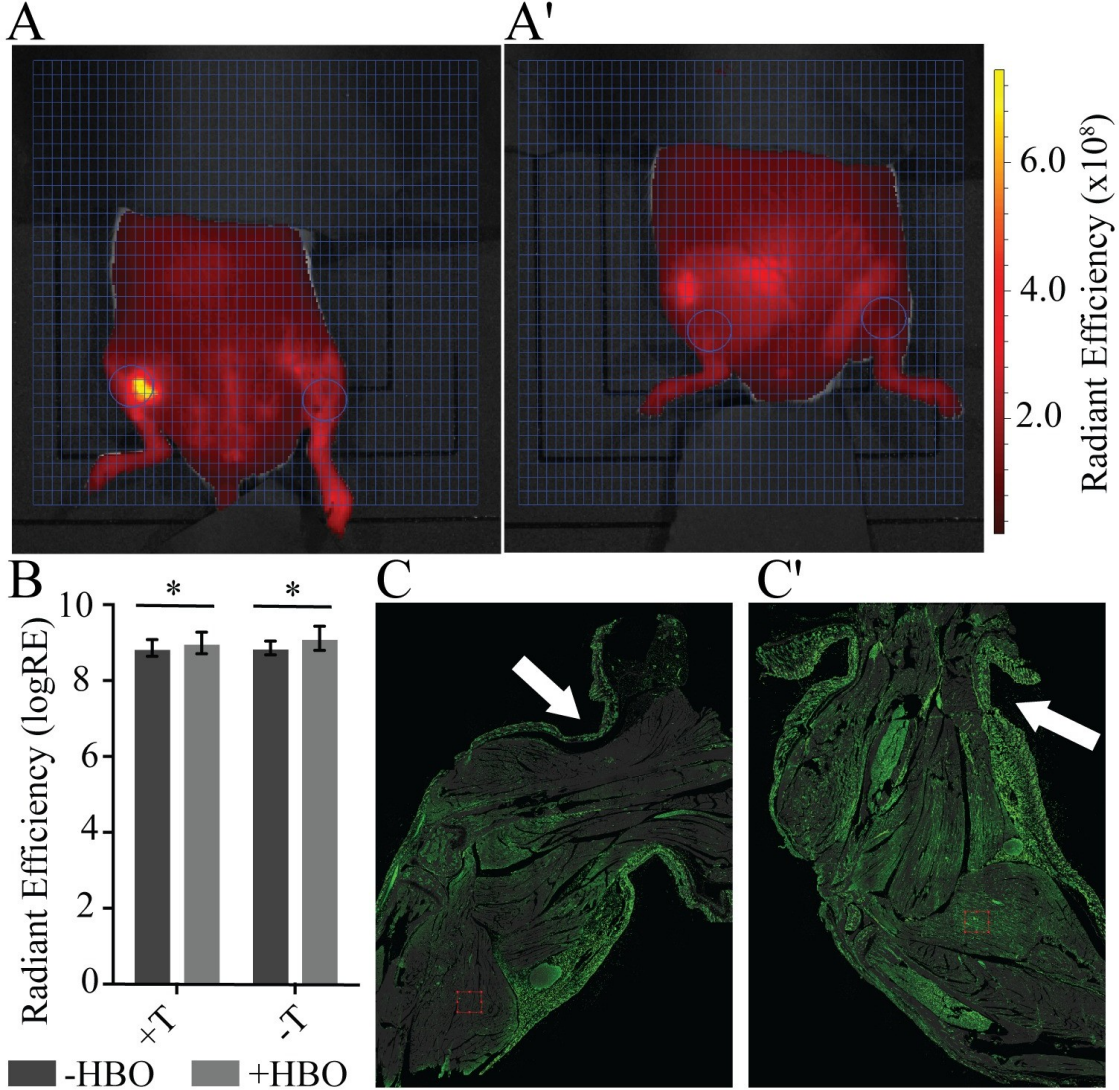
A single application of hyperbaric oxygen (HBO) decreases intracellular hypoxia. Region of interest (ROI—circles) targets the tissue distal to the tourniquet in tourniqueted (L) and untourniqueted (R) limbs for **A)** +HBO and **A')** -HBO. Ventral view. Fluorescent signal indicates Neutrophil Elastase FAST detection (red) in units of radiant efficiency (see scale bar). Representative images shown, N = 8 (-HBO) and 10 (+HBO). **B)** Treatment with HBO increases neutrophil elastase activity in both the tourniqueted and untourniqueted legs, as compared to the -HBO control group. N = 8 (-HBO) and 10 (+HBO). Error bars represent SD**. C)** Intracellular hypoxia (Hypoxyprobe—green) is reduced when HBO is applied while the tourniquet is in place, as compared to **C')** an untreated leg. Hypoxyprobe signal marks cells with <1.3% oxygen. Arrows indicate tourniquet (T) location on limb.

HBO has been shown to decrease the presence [17, 18] of neutrophils and expression of elastase by activated neutrophils [19] after ischemia-reperfusion injury, and is thought to have a protective effect against tissue damage. To determine whether control of tissue damage was due to a local reduction in activated neutrophils we utilized Neutrophil Elastase FAST to identify only activated neutrophils that were producing neutrophil elastase. Surprisingly, quantification of Neutrophil Elastase FAST shows that HBO caused a bilateral increase in neutrophil elastase activity (p = 0.032), suggesting that the reduction in tissue damage is not the result of a decrease in the degradation response from activated neutrophils (Fig 5A and 5B).

HBO supersaturates blood plasma, allowing increased delivery of oxygen beyond the physiological capacity of hemoglobin via the circulatory system [20–23]. To resolve whether the supersaturated residual plasma flow distal to the tourniquet was enough to alleviate ischemia-induced hypoxia in the limb we measured intracellular hypoxia in an untreated and HBO-treated tourniqueted limbs using Hypoxyprobe. A reduction in Hypoxyprobe signal, particularly in the skeletal muscle of the treated leg shows that HBO during tourniquet placement can reduce intracellular hypoxia in the tissue distal to the tourniquet (Fig 5C and 5C').

## Discussion

Mice and swine predictably manifested residual plasma flow distal to an engaged tourniquet in our studies and parallel peer-reviewed studies reporting similar results [6, 7, 24]. For small animals, technology such as the IVIS imager has been used previously in conjunction with ICG to demonstrate blood and plasma flow. However, the limitation of *in vivo* fluorescence imaging to small animals has prevented similar quantification in large animal models and humans in the same capacity. The recent development of portable NIR imaging devices such as the SPY and Fluobeam [25, 26] have allowed us to confirm previous studies on residual plasma flow in swine [6] move beyond prior studies to evaluate human blood flow *in situ*. This technology has demonstrated herein that 70% of human subjects with an extremity tourniquet in place also exhibit residual plasma flow and this flow directly correlated with MAP and BMI in volunteers. In the mouse model of tourniquet-induced limb ischemia we found that supersaturation of the residual plasma flow effectively reduces tissue intracellular hypoxia within central tissue regions and has the potential to play a critical role in protecting ischemic limb tissue from acute damage.

Our findings in human volunteers is particularly interesting, given the high number of individuals with residual flow and overall patterning of the ICG signal. 5 of our 24 volunteers had residual plasma flow that was between 50% (3 volunteers) and 100% (2 volunteers). Our use of ICG in this experiment, which has a relatively short half-life of approximately 180 seconds and binds to proteins in the plasma [27], provided a real-time estimate of plasma containing ICG distal to the tourniquet without the risk of intracellular accumulation [28]. Of note is that, in cases where there is residual flow, while ICG signal distal to the tourniquet increases over time initially (in both the tourniquet and control limb), the subsequent decrease in signal in both limbs supports that volunteers not only had plasma influx but also efflux in tourniqueted limb (S1 Video), and that ICG signal was likely not a result of accumulation of ICG in the ischemic tissue. That being said, we use Doppler initially to confirm the absence of arterial signal and instructed the volunteer to remain completely still, however, we cannot exclude the possibility that volunteers may have shifted in a way that increased residual flow after initial Doppler confirmation. This would increase the fluorescent intensity of the initial baseline reading and necessarily the overall percent flow resulting in 100% flow in the tourniqueted arm.

Given that initial ICG signal is seen in the tips of the fingers before moving proximal towards the tourniquet, and not initially seen in the dermis traveling distally, and that bone receives approximately 10% of cardiac output [29], we hypothesize that plasma flow may be facilitated by central vasculature internal to the bone structure or close to the surface of the bone. This cardiac output to the bone would increase in a linear fashion as MAP increases, at least partially explaining the direct relationship between increased residual flow and MAP. Similarly, increased BMI translates to excess adipose tissue and total blood volume, resulting in increased cardiac output to meet the metabolic demand of the adipose tissue [30–32], which in turn could explain the direct relationship between increased residual flow and BMI.

We tested the therapeutic the utility of this plasma flow by applying hyperbaric oxygen to the tourniqueted limb. In mice, this minimal flow is able to act as a vehicle to penetrate the tourniquet barrier and influence tissue quality while the tourniquet is still in place. The application of hyperbaric oxygen to mice during tourniquet engagement resulted in a significant improvement in tissue quality 48 hours after treatment, long after the oxygen was removed. The immediate downregulation of tissue hypoxia in the limb following treatment suggests that improved tissue salvage is due to a sustained supply of oxygen during tourniquet placement. While the shallow depth of the mouse limb, as compared to the human, makes it difficult to discern whether distribution of ICG in the limb is peripheral in the epidermis or central near to the bone (Fig 1A), the fact that intracellular hypoxia is reduced at the center of the mouse limb, closest to the bone while the epithelium is predominantly hypoxic (Fig 5) suggests that plasma distribution, and necessarily oxygen distribution, is routed more centrally in the limb and parallels our findings in the human volunteers. It should be noted that the effective single 90-minute treatment of HBO utilized here was far below the prolonged levels of HBO exposure that are required to produce oxygen toxicity [33], and that it stands to reason that the pressure, oxygen, treatment length, could possibly be further reduced while remaining effective.

Further supporting our hypothesis that the bone interior can facilitate residual plasma flow, high resolution microCT studies in both mouse and humans describe an extensive network of transcortical capillaries that perpendicularly traverse cortical bone along the shaft to provide continuous blood circulation from the marrow to the periosteum of long bones. Circulation in these systems would be protected during tourniquet application to the exterior of the limb and may serve as a mechanism for plasma flow distal to the tourniquet [34]. Together, MAP, BMI, and individual anatomical differences in transcortical capillary systems may account for differences in flow in both our animal model and the human volunteers. That being said, the limitations of our study did not allow us to investigate what these anatomical differences would be, paving the way for future studies that could further evaluate these differences.

While hemorrhage and exsanguination were not included in the design of this study, given that the purpose of the study was to elucidate characteristics of distal perfusion and cell viability with proximal tourniquet occlusion, our findings demonstrate persistent plasma flow in multiple settings, despite pressures that would normally be thought to prevent any distal blood flow. While the inclusion of both the control and the tourniquet limb in the imaging field of view was critical to the design of the experiment (S1 Video and Fig 3C), we were pleased to find that the elimination of the control limb to decrease the imaging field of view, as would be standard outside the constraints of this experiment, increased ICG detection. This lends support to the idea that future studies incorporating limb injury will benefit from this increased signal.

Confirmation of human plasma flow is particularly relevant given that the tourniquet is treated as a barrier in orthopaedic surgeries. Guidelines for the pharmacologic administration of solutions such as antibiotics address the tourniquet as a barrier and direct that application must be 5 to 10 minutes before tourniquet inflation to allow good tissue penetration [35–37] given that the pharmacokinetics is thought to be limited. The key clinical benefit shown here may be that therapies to support hypoperfused distal tissue during tourniquet application can be considered concurrently. Tourniquets are routinely used during non-emergent elective extremity surgery [3]. The use of LOP with surgery-based tourniquets where a personalized approach to pressure creates a bloodless surgical field has risen in popularity as a way to reduce potential tourniquet-related injuries and avoid unnecessarily high tourniquet pressures [38]. The development of ICG fluorescent angiography has shown that blood flow in injured tissue predicts the extent of tissue damage [39]. More recent studies involving limb trauma have shown that the quantitative utility of ICG can be used to guide subsequent therapy and assist surgical decision making in orthopaedic trauma [40, 41]. Our study, as well as those cited, do not seek to redefine new standard pressures or techniques for tourniquet application, but rather provide roadmaps for future studies that may, or may not, demonstrate that LOP or modified LOP would be useful in elective cases by creating a clinically advantageous plasma flow while achieving a relatively bloodless field appropriate for the procedure.

Plasma flow in conjunction with tourniquet placement is a physiological advantage that has been largely overlooked due to technological obstacles until now. Our findings support the use of preservation techniques concurrent with proper tourniquet placement and determination of minimal LOP based on individual patient parameters. Further, these results provide new consideration for the application of oxygen, tissue preservation solutions, and anti-microbial agents to promote outcomes prior to tourniquet release, all of which are considerations in an environment of increased tourniquet use. Limitations of this investigation include the absence of a hemorrhagic component in the animal and human study arms. Although this investigation focused on the basic physiology of perfusion distal to a tourniquet in a non-trauma setting, we anticipate that these studies set the stage for future clinical research that will include injury models that require tourniquet pressures necessary to occlude distal pulses and to stop wound bleeding, as described in prior military studies [42]. We predict great utility for this technology in elective extremity surgeries that require tourniquets and feel that future studies will uncover additional avenues of limb preservation techniques as the use of tourniquets addresses not only protection of life, but also of limb.

## Supporting information

S1 Video Plasma flow distal to the tourniquet in a human subject. ICG detection in a human subject is evident in both the tourniqueted limb (L) and control limb (R). ICG was injected at T = 20 seconds. Dorsal view.
(MP4)

S1 File Mouse raw data. (XLSX)

S2 File Human raw data. (XLSX)
